# Impact of transparent tray-based application of bioactive glasses desensitizer on the permeability of enamel and dentin to hydrogen peroxide: an in vitro study

**DOI:** 10.1186/s12903-020-01096-5

**Published:** 2020-04-10

**Authors:** Qian Ma, Jingwen Chen, Xiao Xu, Tianda Wang

**Affiliations:** 1 Jiangsu Key Laboratory of Oral Diseases, Nanjing Medical University; Department of General Dentistry, The Affiliated Stomatological Hospital of Nanjing Medical University, Nanjing Medical University, Nanjing, 210029 Jiangsu China; 2 Stomatological College of Nanjing Medical University, Nanjing Medical University, Nanjing, 210029 Jiangsu China

**Keywords:** Bioactive glasses, Dentin hypersensitivity, Permeability, Tooth bleaching, Transparent tray

## Abstract

**Background:**

To investigate the effect of transparent tray-based application of bioactive glasses (BGs) desensitizer on the permeability of enamel and dentin to hydrogen peroxide (H_2_O_2_).

**Methods:**

Freshly extracted human first premolars were divided into 6 groups (*n* = 8). Group A and B: without pretreatments; Group C and E: treated with BGs desensitizer only; Group D and F: treated with BGs desensitizer dispensed with a transparent tray. After roots and pulp tissues of the treated tooth specimens were thoroughly removed, acetate buffer was added into pulp chambers and the treated specimens were immersed in distilled water (Groups A, E, and F) or 30% H_2_O_2_ (Groups B, C, and D) for 30 min at 37 °C. The amount of H_2_O_2_ in the pulp chamber of each group was measured using ultraviolet-visible spectrophotometry.

**Results:**

In control groups (Group A, E, and F), H_2_O_2_ was not detected. The amount of pulpal H_2_O_2_ in Group B, C, and D were 21.149 ± 0.489 μg, 9.813 ± 0.426 μg, and 4.065 ± 0.268 μg respectively. One-way ANOVA analysis indicated that significant differences existed in these groups (F = 459.748, *p* < 0.05).

**Conclusions:**

The effect of BGs desensitizer in reducing the permeability of enamel and dentin to H_2_O_2_ could be enhanced when dispensed with a transparent tray.

## Background

Tooth whitening is any process that lightens the color of teeth. Whitening could be achieved either by physically removing discolored dental hard tissues or chemically bleaching stains. Compared with resin or ceramic veneers and all-ceramic crowns which could physically replace discolored hard tissues, chemically tooth bleaching is considered to be the simplest, most cost-effective and least invasive method of treating colored teeth [1–4]. Therefore, it is now widely applied.

The active ingredient in most tooth bleaching products is peroxide in the form of hydrogen peroxide (H_2_O_2_) or carbamide peroxide. For example, in-office used bleaching products are highly concentrated (35% or 40%) H_2_O_2_ solutions or bleaching gels containing 5.9% of H_2_O_2_. Over-the-counter products are usually liquid gels containing a low concentration (10%) of H_2_O_2_ [1, 3, 4]. Peroxides are highly reactive and could convert to superoxide (O^2−^), hydroxyl (HO-), peroxyl (ROO-) or alkoxyl (RO-) radicals in physical environments. These oxidative oxygen species can freely penetrate dental enamel, react with discolored substances and exert their bleaching or whitening effect. However, side effects like pulp damage would occur when H_2_O_2_ and oxygen species are present at high concentrations or when oral tissues are exposed to them for a long period of time [5]. H_2_O_2_ penetrates dentinal tubules and damages the pulp based on its low molecular weight and the ability to induce protein denaturation [6]. Similarly, the presence of reactive oxygen species in the process of tooth bleaching can lead to temporary inflammations of the pulp and even irreversible changes [7–11].

Since H_2_O_2_ and other reactive oxygen species come into contact with the pulp after penetrating dentinal tubules, attempts to occlude dentinal tubules prior to the application of tooth bleaching products might prevent post-treatment complications from occurring. Hydroxyapatite, the main component of dental hard tissues, is considered as the most ideal material for occluding dentinal tubules. As have been demonstrated in other studies, dentin desensitizers containing bioactive glasses (BGs) could release calcium and phosphorus ions in physiological environments and induce the formation of hydroxyapatite deposit within dentinal tubules [12–14], which could prevent H_2_O_2_ and other reactive oxygen species from penetrating dentinal tubules and reaching the pulp. Hydroxyapatite is considered to have the potential to restore enamel surface, which might also keep the reactive bleaching oxygen species from penetrating the enamel [6]. Moreover, nanoscaled hydroxyapatite contained in a desensitizing paste was demonstrated not to jeopardize its whitening effect [15]. Thus, the application of dentin desensitizers containing BGs prior to tooth bleaching is an ideal method for dealing with post-bleaching complications. However, most dentin desensitizers are administrated by simply smearing on teeth. Due to poor patient compliance and short exposure times, their dentinal tubule occluding effects are limited. Therefore, it is expected that a method capable of improving the dentinal tubule occluding effect of dentin desensitizing agents, particularly before the application of dental bleaching products, can be developed.

The aim of this in vitro study is to investigate the impact of BGs desensitizer on the permeability of enamel and dentin to H_2_O_2_. In the present study, BGs desensitizer was dispensed onto tooth specimens with custom made transparent trays which were commonly used clinically as orthodontic retainers or tooth whitening products containers. Transparent trays here were used in order to obtain enough contact time and an effective concentration of the BGs desensitizer. The null hypotheses were (1) BGs desensitizer had no significant effect on the permeability of dentin and enamel to H_2_O_2_ and (2) customized transparent trays had no significant effect on improving the efficiency of the BGs desensitizer on reducing the amount of H_2_O_2_ infiltrating into pulp chambers.

## Methods

### Tooth preparation

Forty-eight human first premolars without caries, fractures or restorations were freshly extracted due to orthodontic or periodontic reasons from 18 to 40 years old patients and collected with the written informed consent under a protocol approved by the Ethics Committee of Nanjing Medical University (No. PJ 2017–047-001). The teeth were cleaned thoroughly and stored in 0.1% thymol aqueous solution at 4 °C for no more than 1 week prior to use. This research proposal was conducted in full accordance with the World Medical Association Declaration of Helsinki.

### Hydrogen peroxide calibration curve

A 0.5 mg/mL of leucocrystal violet solution was obtained by dissolving 10 mg of leucocrystal violet (Sigma-Aldrich, St. Louis, MO, USA) in 20 mL of distilled water (DW). A 1.0 mg/mL horseradish peroxidase solution was obtained by dissolving 10 mg of horseradish peroxidase (Sigma-Aldrich, St. Louis, MO, USA) in 10 mL of DW. A series of H_2_O_2_ solutions, the concentrations of which were 0.05 μg /mL, 0.10 μg /mL, 0.25 μg/mL, 0.50 μg /mL, 1.0 μg /mL, 2.5 μg /mL, and 5.0 μg /mL, were obtained by diluting 30% H_2_O_2_ stock solution (Fisher Scientific, Pittsburgh, PA, USA) with DW.

To obtain a H_2_O_2_ solution calibration curve, 100 μL of 0.5 mg/mL leucocrystal violet solution and 50 μL of 1.0 mg/mL horseradish peroxidase solution were added into 100 μL, 50 μL, 20 μL, 10 μL, 5 μL, 2.5 μL, and 1.25 μL of H_2_O_2_ solutions and then diluted to 10 mL with acetate buffer (pH = 4.5). The concentrations of H_2_O_2_ in these solutions were 5.0 μg /mL, 2.5 μg/mL, 1.0 μg /mL, 0.5 μg /mL, 0.25 μg /mL, 0.1 μg /mL, and 0.05 μg /mL respectively. After vibrated for 30 s, these solutions were measured with an Ultraviolet-visible (UV-Vis) spectrophotometer (UV-2600, Shimadzu Co., Kyoto, Japan) at a wavelength of 596 nm against a reference prepared using the same method but with no hydrogen peroxide. A calibration curve was thus obtained [16].

### Transparent tray preparation

**Fig. 1:**
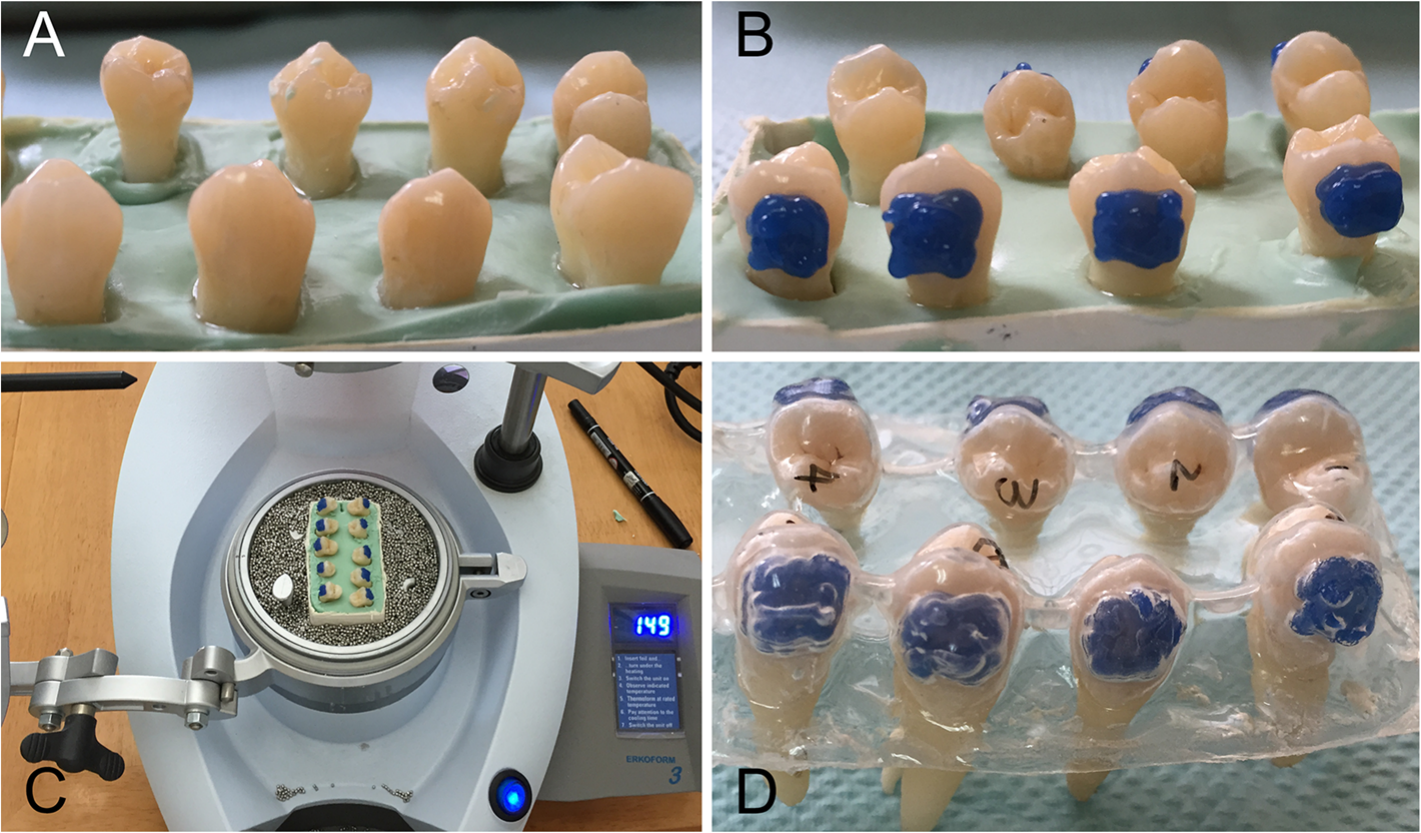
The preparation of customized transparent tray. **a**: Tooth specimens were embedded with alginate impression material with the buccal surfaces exposed; **b**: Gapping discs which were made of light-cured composite resin were placed on buccal reserved areas of the embedded tooth specimens; **c** and **d**: Transparent tray was fabricated with polyvinylacetate plates by a vacuum thermoforming unit at 175 °C on the embedded tooth specimens

Gapping resin discs with a thickness of 1.5 mm which could cover the reserved areas on buccal surfaces of tooth specimens were prepared with a light-cured composite resin (LC Block-Out Resin, Ultradent, South Jordan, UT, USA). This kind of light-cured composite resin was chosen because we didn’t expect an additional dentinal tubule sealing effect. As the surfaces of the tooth specimens were not acid-etched at all and no adhesive was applied, the resin could be easily removed. Therefore, it can hardly lead to a sealing effect. Tooth specimens were embedded with alginate impression material (Jeltrate, Dentsply, Tianjin, China) with the buccal surfaces exposed and the gapping resin discs were placed on buccal reserved areas of tooth specimens (Fig. 1a and b). Transparent trays were then fabricated with 1.0-mm polyvinylacetate plates (Erkoflex, Erkodent Erich Kopp GmbH, Pfalzgrafenweiler, Germany) using a vacuum thermoforming unit (Erkoform-3d, Erkodent Erich Kopp GmbH, Pfalzgrafenweiler, Germany) at 175 °C on these tooth specimens (Fig. 1c and d).

### Experimental procedures

Tooth specimens were randomly divided into 6 groups and each group contained 8 teeth.

**Fig. 2:**
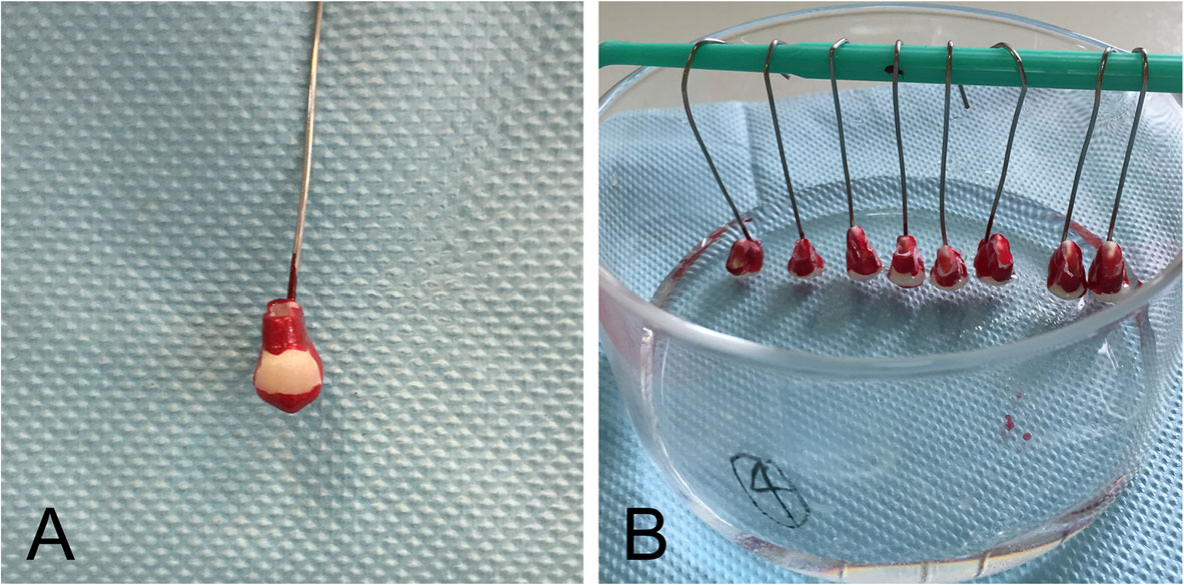
The treatment of tooth specimens in Group A and Group B. **a**: The crown surfaces of experimental tooth specimens were coated with 2 layers of acidproof varnishes, except a reserved area on buccal surface 1 mm away from CEJ; **b**: After turned upside down with the occlusal surface pointed downwards and 25 μL of 2 mol/L acetate buffer added into the pulp chamber, the tooth specimens were immersed in DW or 30% H_2_O_2_ solution for 30 min at 37 °C with the level of the liquid lower than the cut surfaces of the teeth

Group A and B: Tooth specimens were cut perpendicularly to the long axis of the teeth 3 mm apically away from the cementoenamel junction (CEJ) with a diamond bur before roots and pulp tissues were thoroughly removed. These tooth specimens were then rinsed with DW for 30 s and air-dried for 15 s. For each tooth, the crown surfaces were coated with 2 layers of acidproof varnishes, except a reserved area on the buccal surface 1 mm away from CEJ (Fig. 2a). After turned upside down with the occlusal surface pointed downwards and 25 μL of 2 mol/L acetate buffer added into the pulp chamber, the tooth specimens were immersed in DW (Group A) or 30% H_2_O_2_ solution (Group B) for 30 min at 37 °C with the level of the liquid lower than the cut surfaces of the teeth (Fig. 2b).

Group C and E: A reserved area 1 mm away from CEJ on the buccal surface of each tooth was treated with a BGs desensitizer (DDS™, Datsing Bio-tech Co., Ltd., Beijing, China) for 2 min, twice with an interval of 12 h. The teeth were rinsed with DW for 15 s after each treatment. After immersed for 24 h in artificial saliva (AS) which contained CaCl_2_ (0.7 mM/L), MgCl_2_•6H_2_O (0.2 mM/L), KH_2_PO_4_ (4.0 mM/L), KCl (30 mM/L), NaN_3_ (0.3 mM/L) and HEPES buffer (20 mM/L) [17], the treated buccal surfaces of these tooth specimens were rinsed with DW for 15 s and the root and pulp tissue of each tooth specimen were removed, followed with DW rinsing for 30 s and air-dried for 15 s. For each tooth, the crown surfaces were coated with 2 layers of acidproof varnishes, except the treated area on the buccal surface. Tooth specimens were turned upside down and 25 μL of 2 mol/L acetate buffer was added into each pulp chamber. The tooth specimens were then immersed in DW (Group E) or 30% H_2_O_2_ solution (Group C) for 30 min at 37 °C with the liquid level lower than the cut surfaces.

Group D and F: A reserved area 1 mm away from CEJ on the buccal surface of each tooth was treated with a BGs desensitizer (DDS™, Datsing Bio-tech Co., Ltd., Beijing, China) dispensed in a transparent tray for 1 h and rinsed with DW for 15 s. After immersed in AS for 24 h, the treated buccal surfaces of the tooth specimens were rinsed with DW for 15 s. Then, the root and pulp tissue of each tooth were thoroughly removed, and the pulp chamber was rinsed with DW for 30 s and air-dried for 15 s. For each tooth, the crown surfaces were coated with 2 layers of acidproof varnishes, except the treated buccal area. Tooth specimens were turned upside down and 25 μL of 2 mol/L acetate buffer was added into each pulp chamber. The tooth specimens were then immersed in DW (Group F) or 30% H_2_O_2_ solution (Group D) for 30 min at 37 °C with the liquid level lower than the cut surfaces.

After treatments mentioned above, acetate buffer in pulp chamber of each treated tooth was transferred into glass tubes labeled as Groups A to F. The pulp chamber of each tooth was rinsed twice with 100 μL of DW which was then transferred to the corresponding glass tubes. One hundred microliters of leucocrystal violet solution (5 mg/mL) and 50 μL of horseradish peroxidase solution (1 mg/mL) were added to each glass tube. After diluted to 10 mL with acetate buffer (pH = 4.5) and vibrated for 30 s, the liquid in each glass tube was measured with UV-Vis spectrophotometer at the wavelength of 596 nm. The amount of H_2_O_2_ in each sample was calculated using the calibration curve.

### Statistical analysis

Statistical analysis was performed using SPSS 13.0 for Windows (SPSS Inc., Chicago, IL, USA). Differences in the amount of H_2_O_2_ in each group were analyzed using one-way ANOVA analysis. Once overall differences among the means were observed, post hoc tests of LSD (least significant difference) were performed for multiple comparisons. *P*-values less than 0.05 were considered statistically significant.

## Results

**Fig. 3:**
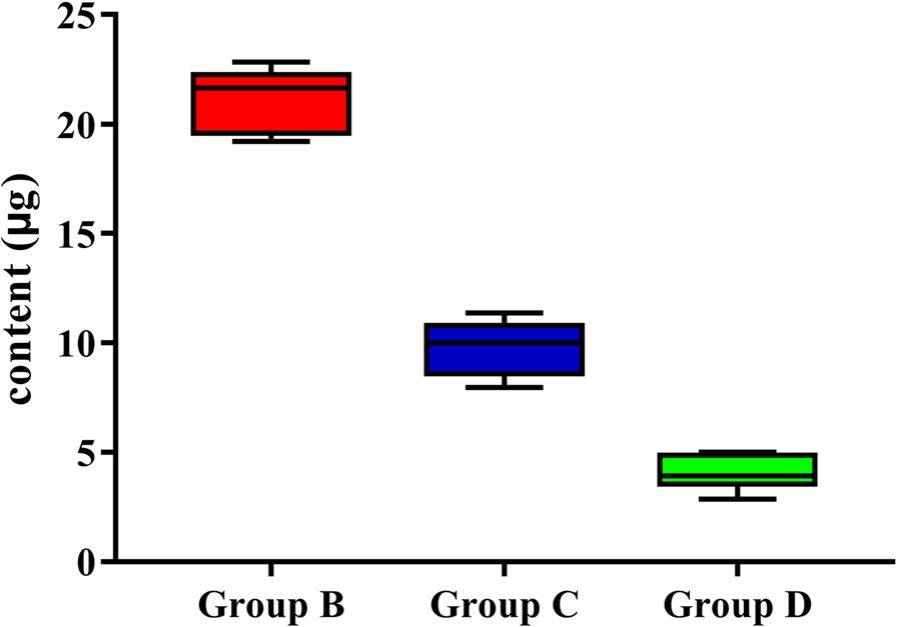
The amount of pulpal H_2_O_2_ in Group B, Group C, and Group D. After tooth specimens were immersed in 30% H_2_O_2_ for 30 min at 37 °C, 21.149 ± 0.489 μg, 9.813 ± 0.426 μg and 4.065 ± 0.268 μg of pulpal H_2_O_2_ was detected in Group B, Group C, and Group D, respectively

As is shown in Table 1, after tooth specimens were immersed in DW for 30 min at 37 °C, regardless of what kind of surface treatment was applied, hydrogen peroxide was not detected in pulp chambers in control groups (Group A, E, and F). In experimental groups (Group B, C, and D), on the other hand, pulpal H_2_O_2_ was found. The amount of pulpal H_2_O_2_ in Group B, Group C, and Group D are shown in Fig. 3. The results one-way ANOVA analysis and LSC post hoc tests indicated that significant differences existed between these groups (F = 459.748, *p* < 0.05).

**Table 1:** The amount of pulpal H_2_O_2_ in each group (μg)

Group	N	Treatments	H_2_O_2_ content (μg)^a^
A (Negative control)	8	No surface treatment, immersed in DW	0.0
B (Positive control)	8	No surface treatment, immersed in 30% H_2_O_2_	21.149 ± 0.489
C	8	Surface treated with BGs desensitizer only, immersed in 30% H_2_O_2_	9.813 ± 0.426
D	8	Surface treated with BGs desensitizer and transparent tray, immersed in 30% H_2_O_2_	4.065 ± 0.268
E	8	Surface treated with BGs desensitizer only, immersed in DW	0.0
F	8	Surface treated with BGs desensitizer and transparent tray, immersed in DW	0.0

^a^ Values are means ± standard deviations

## Discussion

Peroxides such as hydrogen peroxide and carbamide peroxide used to bleach discolored dental hard tissues have been shown to have the ability to penetrate enamel and dentin, which is determined by several factors including the concentration of peroxides, the exposure time of teeth to peroxides, cracks and restorative materials existing on teeth, and etc. [18–23]. As was shown in previous studies, more than 66% of patients suffered from dentin hypersensitivity or gingival irritation after tooth bleaching [9]. Other studies have also reported that the infiltration of peroxides into the pulp could lead to pulpal inflammation and necrosis [24, 25]. Although at present, no literature has given the concentration of H_2_O_2_ in the pulp that can lead to dentin hypersensitivity of post-bleaching pain of patients, in a study using bovine teeth, bleaching agents using H_2_O_2_, the concentrations of which were 20 and 35%, as their main components were shown to lead to moderate or severe inflammation in the upper thirds of the coronal pulp. The authors concluded that in-office bleaching protocols using lower concentrations of H_2_O_2_ should be preferred [26]. Since tooth bleaching agents are thought to infiltrate into the pulp through dentinal tubules, the application of dentin desensitizers which could occlude dentinal tubules is, therefore, an effective option to help dentists deal with dentin hypersensitivity and pulpal damage emerging during or after tooth bleaching [27–29]. Dentin desensitizers containing potassium nitrate or sodium fluoride are widely used to solve dentin hypersensitivity, especially before or after tooth bleaching. They are also added into bleaching agents to alleviate pulpal responses without jeopardizing the tooth bleaching effect. However, desensitizing agents which contain potassium nitrate relief pulpal symptoms primarily by reducing the activity of dentinal sensory nerves, which is the main effect of potassium ions [30]. As a result, they cannot help us prevent the infiltration of peroxides into pulp tissues from occurring or reduce the amount of pulpal peroxides. Thus, they are not ideal for us to solve post-bleaching pulpal damage. Sodium fluoride, on the other hand, alleviates dentin hypersensitivity by inducing a calcium fluoride layer which covers enamel and exposed dentin [30]. However, as this layer could be easily removed by tooth brushing, the utilization of sodium fluoride is limited. Therefore, it is urgent that better products or methods be developed.

Bioactive glasses (BGs), the main component of which is CaO-Na_2_O-P_2_O_5_-SiO_2_, were developed in Professor Hench’s laboratory at the University of Florida in 1969 and were later known as 45S5 or Bioglass® [31]. Due to the ability in inducing the formation of hydroxycarbonate apatite (HCA) in physiological conditions, BGs are comprehensively studied in the fields of tissue engineering, bone regeneration and dentin remineralization [31–33]. BGs have been demonstrated to occlude dentinal tubules by inducing hierarchical mineralization via the precipitation of apatite composed of calciumsilicate in dentinal tubules [34]. Our previous study showed that dentinal tubules, which were in tooth specimens treated twice a day with BGs desensitizing paste and immersed in artificial saliva for 7 days, could be completely occluded with calcium-deficient hydroxyapatite deposits to a depth of 5.5–8.1 μm [14]. Therefore, BGs desensitizers would be ideal to alleviate dentin hypersensitivity, especially when it happens during or after the process of tooth bleaching.

Although BGs desensitizer is considered to be an ideal material to treat dentin hypersensitivity, in clinical practice, most dentin desensitizers including BGs desensitizing paste are administrated by patients themselves according to the manufacturer’s instructions after the first application carried out by dental practitioners. Poor patient compliance and short therapeutic exposure will lead to relatively limited dentinal desensitization and dentinal tubule occlusion effects. In order to deal with this condition, a transparent tray which could hold the desensitizer for a while in patients’ oral cavities is considered. In our previous research, better dentinal tubules occluding effect could be guaranteed by using BGs desensitizer dispensed in a transparent tray [14]. Therefore, a more effective approach to deal with dentin hypersensitivity and pulpal damage during or after tooth bleaching is expected. In the present study, the peroxides penetration-reducing effect of BGs desensitizer in the existence of transparent trays was explored by measuring the amount of pulpal H_2_O_2_ through UV-Vis spectrophotometry after pretreated tooth samples were immersed in 30% H_2_O_2_.

As is shown in Table 1, no pulpal H_2_O_2_ was detected in teeth in group A, E, and F. The concentration of pulpal H_2_O_2_ in teeth without BGs desensitizer treatment (group B) was the highest among all experimental groups. This fact, which was very similar to that reported by other researchers, indicated that H_2_O_2_ could penetrate enamel and dentin, as was assumed before. Concentrations of pulpal H_2_O_2_ in tooth specimens treated with BGs desensitizer, regardless of whether transparent trays were employed or not, were significantly lower than that in untreated tooth specimens, indicating that BGs desensitizer had the ability to keep H_2_O_2_ from infiltrating into pulp chambers. This could attribute to the dentinal tubule occluding effect of HCA deposits which were induced by BGs in artificial saliva. A series of stages were thought to be responsible for HCA formation in physiological fluids including artificial saliva. Firstly, rapid ion exchange happens between Na^+^ and Ca^2+^ from BGs and H^+^ from the solution, leading to the formation of silanol bonds and a silica-rich layer on the surface of BGs. Thereafter, an amorphous calcium phosphate layer on the surface of this silica-rich layer appears by sequestering Ca^2+^ and phosphorous and further crystallizes into HCA layers, consuming OH^−^ and CO_3_^2−^. HCA layers then combine with each other and with dentinal tubule walls, leading to their dentinal tubule occluding effect [35–37]. HCA layers are also believed to restore cracks within dental enamel.

In this study, transparent trays were demonstrated to help improve the effect of BGs desensitizer as the concentration of pulpal H_2_O_2_ was significantly decreased in BGs desensitizer treating groups after transparent trays were employed. Customized trays are widely used as removable retainers in orthodontics and also as agent-holding devices using in tooth bleaching [38]. In the latter condition, customized trays have been found to reduce the penetration of peroxide into the pulp chamber [39]. Moreover, clinical trials have demonstrated that the level of sensitivity during the in-office tooth bleaching procedure could be reduced when bleaching agents were covered with customized trays [40, 41]. Here, custom-fitted transparent trays were used as the supporter of BGs desensitizer in order to prolong the treatment duration by putting BGs desensitizer into the storage space [32, 42, 43]. By doing so, the dilution of BGs desensitizer by saliva in patients’ oral cavities would be decelerated and the exposure duration of tooth to desensitizer would be extended.

## Conclusion

With the limits of this study, the null hypothesis has to be rejected. The additional use of transparent trays, combined with a simple coating of BGs containing dentin desensitizer, could significantly reduce the infiltration of H_2_O_2_ into pulp chambers. In conclusion, application of BGs desensitizer with transparent trays as its container was considered to be more effective in keeping peroxides in tooth bleaching agents from penetrating enamel and dentin and damaging pulp tissues during or after tooth bleaching treatment. This could open up new possible applications in future clinical dentistry.

## Data Availability

The data sets used and analyzed during the current study are available from the corresponding author upon reasonable request.

## Abbreviations

H_2_O_2_: Hydrogen peroxide
BGs: Bioactive glasses
DW: Distilled water
UV-Vis: Ultraviolet-visible
CEJ: Cementoenamel junction
AS: Artificial saliva
HCA: Hydroxycarbonate apatite
